# The Clinical Effect of Arthroscopic Rotator Cuff Repair techniques: A Network Meta-Analysis and Systematic Review

**DOI:** 10.1038/s41598-019-40641-3

**Published:** 2019-03-11

**Authors:** Binwu Xu, Long Chen, Jun Zou, Yurong Gu, Liang Hao, Kun Peng

**Affiliations:** 1 Department of Orthopedics, the second affiliated hospital of Nanchang university, Nanchang, Jiangxi China; 2 Department of Orthopedics, Guizhou Provincial People’s Hospital, Guiyang, Guizhou China; 3 Department of Orthopedics, Jiangxi Provincial Children’s Hospital, Nanchang, Jiangxi China

## Abstract

Rotator cuff tears are common and are associated with shoulder pain, disability, and dysfunction. Previous studies that have reported different arthroscopic techniques are controversial. A network meta-analysis with indirect and direct evidence was performed to compare the effectiveness of arthroscopic techniques for the treatment of rotator cuff tears. PUBMED, the Cochrane Register of Controlled Trials, EMBASE and Scopus were searched based on the Preferred Reporting Items for Systematic Reviews and Meta-Analyses (PRISMA) statement, and related studies that evaluated arthroscopic techniques for the treatment of rotator cuff tears were identified in May 2018. The primary outcome measure was the retear rate. The secondary outcome measures included the constant score and the range of motion (forward flexion and external rotation). Twenty-one trials comprising 1815 shoulders were included in the study. This study indicated that single-row (SR) repair resulted in a higher retear rate than suture bridge (SB) and double-row (DR) repairs. Moreover, the SR and DR repairs resulted in higher incidences of retear than SB repair. The ranking of the treatments based on the constant score and external rotation was SB repair, SR repair and DR repair, whereas the treatment ranking according to forward flexion was SB repair, DR repair and SR repair. In summary, this network meta-analysis provides evidence that SB repairs might be the best choice to improve the postoperative recovery of function and decrease the retear rate.

## Introduction

Rotator cuff tears are among the most common shoulder injuries and are usually accompanied by shoulder pain, disability, and dysfunction^1,2^. Previous studies have reported that approximately 25% of sixty-year-olds and 45% of seventy-year-old patients experience difficulties in daily living^3–5^. Over the past decade, rotator cuff repairs have shifted from open to arthroscopic techniques, and the arthroscopic technique is associated with less morbidity than the open technique and yields comparable clinical results^2,6^.

The most commonly used arthroscopic techniques are the single-row (SR), suture bridge (SB) and double-row (DR) repairs. Despite the popularity of arthroscopic rotator cuff repair, no consensus exists regarding the preferred fixation method. Previous studies have demonstrated that DR repair decreases gap formation and increases mechanical strength, footprint coverage, and watertight isolation of the healing zone interface from the synovial fluid environment^7^. SR repair results in lower implant costs and a reduced blood supply^8,9^. The SB repair, occasionally called the “transosseous-equivalent (TOE)” repair, is an alternate DR technique that has been biomechanically shown to result in a greater tendon-bone contact area, a higher tendon-bone contact pressure, and a higher load to failure than conventional DR^10–13^. Some reivews previously reported that SR repair resulted in a higher retear rate than DR repair, DR repair provides greater external rotation and no significant difference was found between SR repair and DR repair in terms of the constant score^8,14^. Howerver, previous pairwise meta-analyses^7,8^ have provided only pairwise comparisons and partial information and do not inform optimal decisions regarding a variety of different treatments for arthroscopic rotator cuff repair^15^. The network meta-analysis could combine indirect and direct evidence to compare the relative advantages of multiple treatments and obtain the recommended level of evidence for selecting clinical options^16^.

The relative efficacies of SR repair, DR repair and SB repair for rotator cuff tears in patients were compared using both network meta-analyses and pairwise meta-analyses to provide recommendations based on the comparative retear rate, constant score, external rotation and forward flexion.

## Methods

This systematic review was designed and performed with an a priori protocol (PROSPERO 42017071720) established according to the Preferred Reporting Items for Systematic Reviews and Meta-Analyses (PRISMA) statement for network meta-analyses and systematic reviews for healthcare interventions^17^.

### Data sources and searches

The PUBMED (from Jan 1980 to May 2018), Cochrane Register of Controlled Trials (May 2018), EMBASE (from Jan 1980 to May 2018) and Scopus (May 2018) databases were searched. All related studies on the efficacy and safety of SR repair, DR repair and SB repair based on the following search terms were collected: (rotator cuff tear) AND (therapy OR surgery OR treatment OR complications OR adverse effect) AND clinical trial; (Rotator Cuff Injuries/adverse effect [Mesh] OR Rotator Cuff Injuries/surgery [Mesh] OR Rotator Cuff Injuries/treatment [Mesh]) AND clinical trial[ptyp].

### Inclusion and exclusion criteria

The inclusion criteria were as follows: (1) patients were diagnosed with rotator cuff tears; (2) the interventions included SR repair, DR repair and SB repair; and (3) the studies were RCTs or retrospective or prospective comparative trials.

The exclusion criteria were as follows: (1) patients with fractures, secondary surgeries, dislocations and other diseases that affect the function of the shoulder; (2) interventions including conventional open or mini-open repair techniques; and (3) case reports.

### Data extraction and quality assessments

The country, study design, patient sample size, different interventions and lengths of follow-up of the included studies were collected. Additionally, the primary outcomes were the retear rate, constant score, external rotation and forward flexion. Two researchers extracted the above data independently, and disagreements were resolved by discussion.

The Cochrane Collaboration tool^18^ was used to assess the risk of bias for the RCTs, and the Newcastle-Ottawa Scale^19^ was used to judge the quality of prospective and retrospective comparative trials. For RCTs, data were obtained regarding random generation, allocation concealment, blinding of the outcomes, incomplete outcomes, selective reporting and other biases^18^. For case-control studies, selection, comparability and exposure were obtained. A total score based on the Newcastle-Ottawa Scale was calculated as presented in Table S1^19^.

### Outcome assessment

The primary outcome for analysis was the retear rate. The secondary outcome measures included the constant score and the range of shoulder motion (external rotation and forward flexion).

### Data synthesis and statistical analysis

Conventional meta-analyses were performed for all primary and secondary outcomes using a random effects model with Stata software (version 13.0). The pooled estimates of the odds ratios (ORs) or standardized mean differences (MDs) and 95% confidence intervals (CIs) of the four outcomes (i.e., retear rate, constant score, external rotation and forward flexion) are presented^20^. Heterogeneity was assessed by chi-square tests and I-square tests. Significance values less than 0.10 for chi-square tests or more than 50% for I-square tests were interpreted as evidence of heterogeneity. Egger’s linear regression test were used for detectint the publication bias with Stata. Network meta-analyses using indirect and direct evidence based on a frequentist framework including network plot, forest plot and predictive interval plot were performed with Stata software (version 13.0). The codes, model and network graphs package used in this meta-analysis are free online^21,22^. The surface under the cumulative ranking curve (SUCRA) was used to determine the ranking of the three arthroscopic rotator cuff repair techniques in terms of rotator cuff retear^23^. The SUCRA was used to measure the therapies; 100% indicates the best treatment, and 0% indicates the worst treatment^23^. Dias model was used to assess the inconsistencies in this study^24^. Fixed effects models and random effects models were used to assess the sensitivity analyses.

## Results

### Study selection

**Figure 1:**
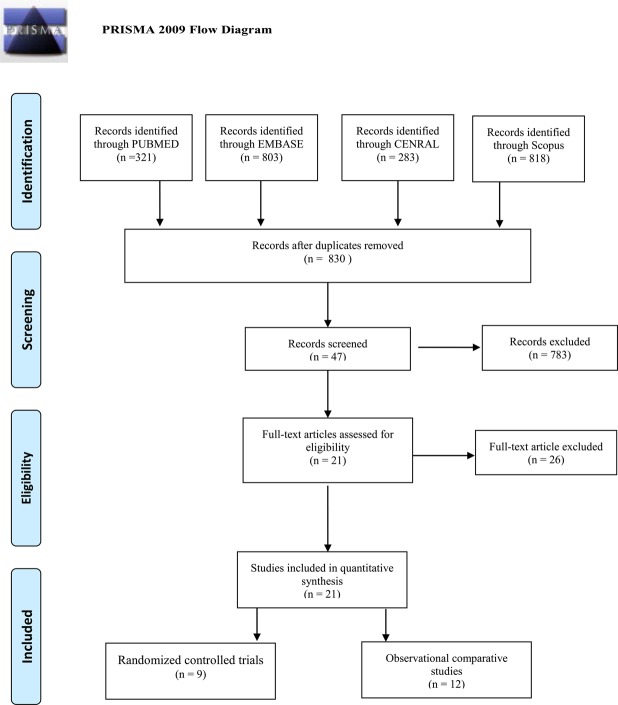
Selection flow of the studies included in the meta-analysis.

The study selection flow is presented in Fig. 1 and was conducted according to the PRISMA statement. A total of 1407 studies (283, 321, 803 and 818 from the Cochrane Register of Controlled Trials, PUBMED, EMBASE and Scopus, respectively) were originally included in this study. After two researchers read the full-texts, twenty-one studies^6,25–44^ (nine RCTs^6,25–27,29,30,34,35,38^ and twelve observational comparative studies^28,31–33,36,37,39–44^) were considered relevant and included in this meta-analysis.

### Characteristics and qualities of the included studies

**Figure 2:**
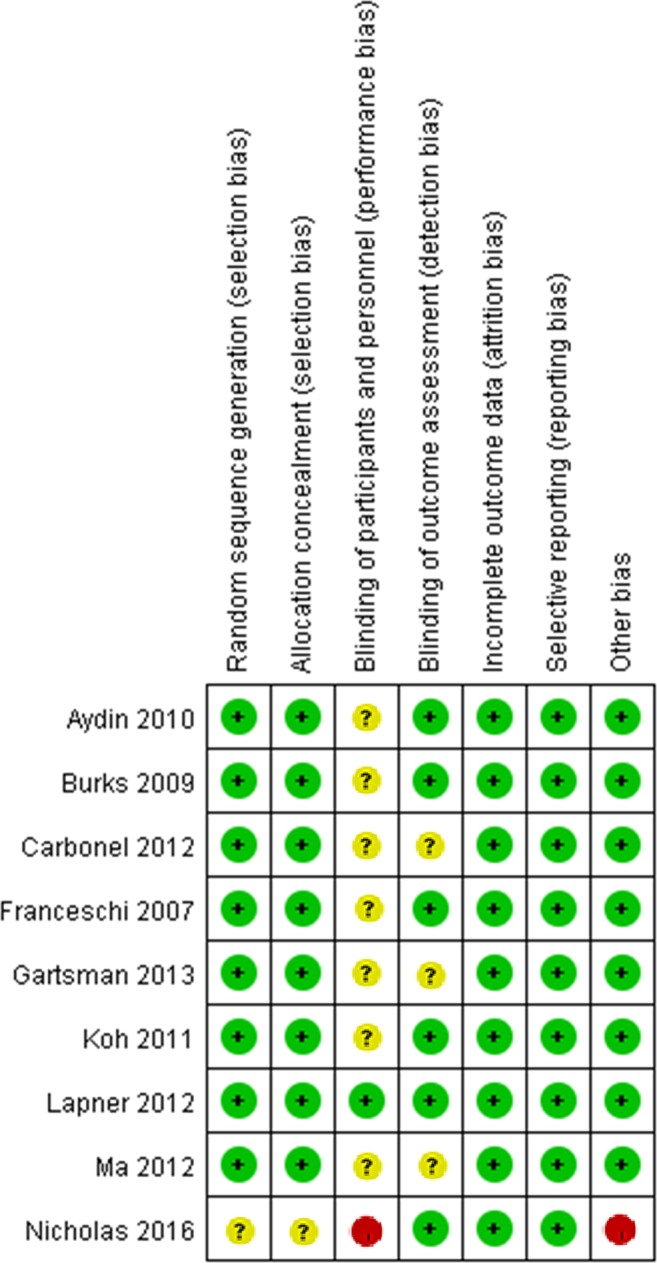
Risk of bias summary for each RCT.

Twenty-one trials containing 1815 shoulders with evaluated treatments were included in the network meta-analysis. The characteristics of the included studies are presented in Table 1. Of the nine randomized controlled trials (RCTs) analyzed, the Cochrane Collaboration tool indicated that eight studies^6,25–27,29,30,34,35^ used adequate randomization, eight studies^6,25–27,29,30,34,35^ used adequate allocation concealment, and six studies^25,26,29,34,35,38^ reported outcome assessment blinding. However, only one study^35^ reported patient blinding. All included studies were free of selective and incomplete outcome reporting. Only one study^38^ had a high risk of other types of bias (Fig. 2). Of the observational comparative studies, three studies^36,39,41^ received eight points, and nine studies^28,31–33,37,40,42–44^ received seven points (Table 2).

**Table 1:** Characteristics of included studies comparing different arthroscopic techniques for rotator cuff repair.

Study	Country	Interventions	Sample size (shoulder)	Follow-up (months)	Study design	Factor analyzed
Aydin 2010	Turkey	SR vs DR	34/34	24	RCT	Constant score
Burks 2009	USA	SR vs DR	20/20	12	RCT	Constant score; Retear rate
Carbonel 2012	Spain	SR vs DR	80/80	24	RCT	Retear rate
Charousset 2007	France	SR vs DR	35/31	24	PC	Constant score; Retear rate
Franceschi 2007	Italy	SR vs DR	30/30	24	RCT	Forward flexion; Retear rate
Gartsman 2013	USA	SR vs SB	40/43	12	RCT	Retear rate
Gerhardt 2012	Germany	SR vs SB	20/20	20	RC	Constant score; Retear rate
Ide 2015	Japan	SR vs SB	25/36	57.5	RC	External rotation; Forward flexion
Kim 2012	Korea	DR vs SB	26/26	24	PC	Constant score; External rotation; Forward flexion; Retear rate
Koh 2011	Korea	SR vs DR	31/31	31.9	RCT	Constant score; External rotation; Forward flexion; Retear rate
Lapner 2012	Canada	SR vs DR	48/42	24	RCT	Constant score
Ma 2012	China	SR vs DR	27/26	24	RCT	Retear rate
Mccormick 2014	USA	SR vs DR vs SB	20/21/22	24	RC	Constant score
Mihata 2011	Japan	SR vs DR vs SB	65/23/107	38.5	PC	Retear rate
Nicholas 2016	USA	SR vs DR	20/16	26.4	RCT	External rotation; Forward flexion
Panella 2016	Italy	SR vs SB	24/20	24	RC	Constant score
Park 2013	Korea	DR vs SB	55/119	24	RC	Constant score
Shin 2015	Korea	SR vs SB	47/37	32.5	RC	Constant score; Retear rate
Sugaya 2005	Japan	SR vs DR	39/41	35	RC	Retear rate
Wade 2017	India	SR vs DR	28/28	6	PC	Retear rate
Wang 2015	China	SR vs DR	146/102	24	RC	Constant score; External rotation; Forward flexion; Retear rate

RCT: randomized controlled trial; RC: retrospective comparative; PC: prospective comparative; SR: single-row repair; DR: double-row repair; SB: suture bridge repair.

**Table 2:** Quality assessment of case-control studies comparing different techniques for arthroscopic rotator cuff repair using the Newcastle-Ottawa Scale.

Author group	Selection				Comparability		Exposure	
	Adequate case definition	Representativeness of the cases	Selection of controls	Definition of controls	Comparability of cases and controls	Ascertainment of exposure	Same method of ascertainment	Non-response rate
Charousset 2007	1	1	—	1	2	1	1	—
Gerhardt 2012	1	1	—	1	1	1	1	1
Ide 2015	1	1	—	1	1	1	1	1
Kim 2012	1	1	—	1	2	1	1	—
Mccormick 2014	1	1	—	1	1	2	1	1
Mihata 2011	1	1	—	1	1	1	1	1
Panella 2016	1	1	—	1	1	2	1	1
Park 2013	1	1	—	1	1	1	1	1
Shin 2015	1	1	—	1	1	2	1	1
Sugaya 2005	1	1	—	1	1	1	1	1
Wade 2017	1	1	—	1	1	1	1	1
Wang 2015	1	1	—	1	1	1	1	1

### Retear rate

**Figure 3:**
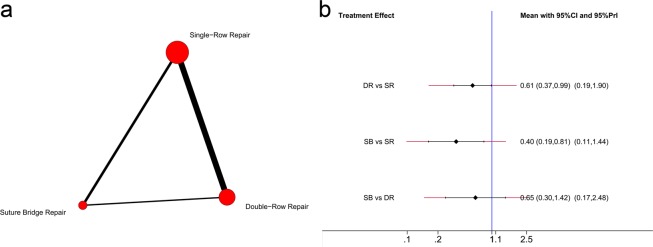
(**a**) Network plot of treatment comparisons for retear rate. The size of the red area indicates the sample size of each group, and the thickness indicates the results of comparisons between two groups; (**b**) The forest plot and predictive interval plot of network meta-analysis for retear rate. SR: Single-Row Repair; DR: Double-Row Repair; SB: Suture Bridge Repair.

**Figure 4:**
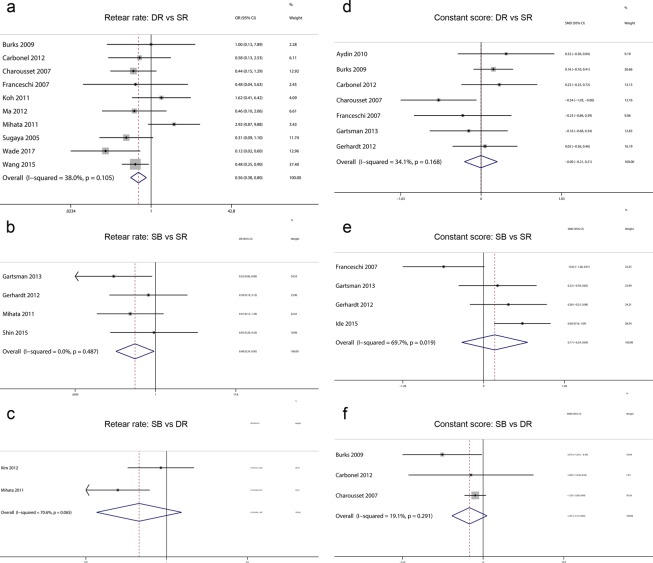
The forest plot of pairwise meta-analysis for retear rate and constant score. SR: Single-Row Repair; DR: Double-Row Repair; SB: Suture Bridge Repair.

Regarding the retear rate, 14 trials^6,26–31,33,34,37,41–44^ were included in this network meta-analysis. A total of 608 shoulders underwent SR repair, 437 underwent DR repair, and 232 underwent SB repair. The network comparisons for the retear rate are presented in Fig. 3a. The effect size (OR with 95% CI) of the retear rate via direct comparison by pairwise meta-analysis is presented in Fig. 4a–c, The effect size (OR with 95% CI and 95% Prl) of the retear rate vianetwork meta-analysis is presented in Fig. 3b. Both the network meta-analysis and pairwise meta-analysis indicated that SR repair resulted in a higher retear rate than the SB [network: 0.40(0.19, 0.81); pairwise: 0.48(0.24, 0.95)] and DR [network: 0.61(0.37, 0.99); pairwise: 0.56(0.38, 0.80)] repairs. No significant difference was found between the DR and SB repairs with regard to the retear rate. Based on the SUCRA probability, SB repair had the lowest probability for retear (0.910), followed by DR repair (0.575) and SR repair (0.016).

### Constant score

**Figure 5:**
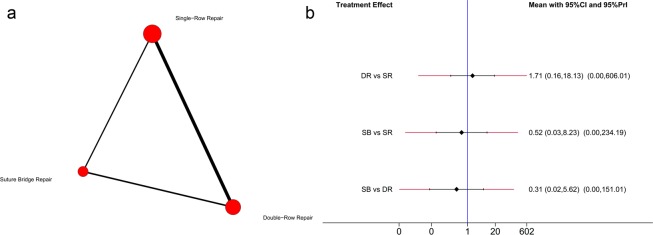
(**a**) Network plot of treatment comparisons for constant score. The size of the red area indicates the sample size of each group, and the thickness indicates the results of comparisons between two groups; (**b**) The forest plot and predictive interval plot of network meta-analysis for constant score. SR: Single-Row Repair; DR: Double-Row Repair; SB: Suture Bridge Repair.

Regarding the constant score, 12 trials^25,26,28,31,33–36,39–41,44^ including 1031 shoulders reported constant score data that met the criteria for inclusion in this network meta-analysis. The network used to compare the constant scores is illustrated in Fig. 5a. Hierarchies of effect size (MD with 95% CI) based on pairwise meta-analysis is presented in Fig. 4d–f. Hierarchies of effect size (MD with 95% CI and 95% Prl) based on network meta-analysis is presented in Fig. 5b. Both pairwise meta-analysis and network meta-analysis indicated no significant differences among SR repair, DR repair and SB repair in terms of the constant score. Based on the SUCRA probability, the ranking from first to third was SB repair (0.720), SR repair (0.539) and DR repair (0.242).

### External rotation

**Figure 6:**
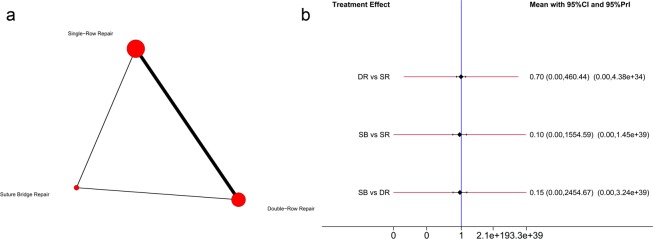
(**a**) Network plot of treatment comparisons for external rotation. The size of the red area indicates the sample size of each group, and the thickness indicates the results of comparisons between two groups; (**b**). The forest plot and predictive interval plot of network meta-analysis for external rotation. SR: Single-Row Repair; DR: Double-Row Repair; SB: Suture Bridge Repair.

**Figure 7:**
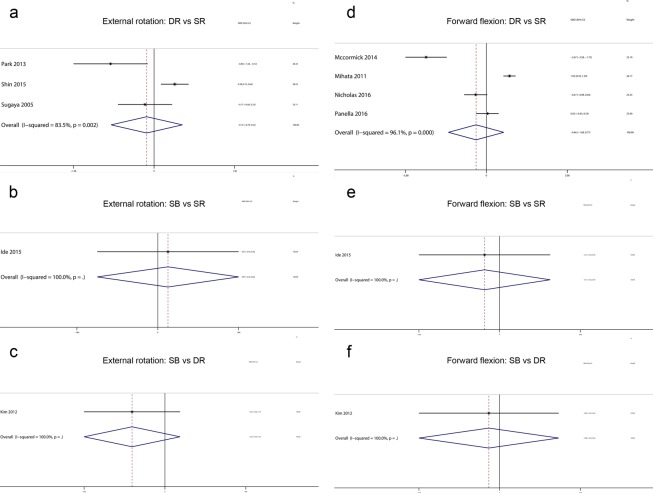
The forest plot of pairwise meta-analysis for external rotation and forward flexion. SR: Single-Row Repair; DR: Double-Row Repair; SB: Suture Bridge Repair.

Regarding external rotation, five trials^32–34,38,44^ were included in this network meta-analysis. The analysis of the comparisons of external rotation is presented in Fig. 6a. Hierarchies of effect size (MD with 95% CI) based on pairwise meta-analysis is presented in Fig. 7a–c. Hierarchies of effect size (MD with 95% CI and 95% Prl) based on network meta-analysis is presented in Fig. 6b. Both pairwise meta-analysis and network meta-analysis indicated no significant differences among SR repair, DR repair and SB repair in terms of external rotation. Based on the SUCRA probability, the ranking from first to third was SB repair (0.700), SR repair (0.425) and DR repair (0.376).

### Forward flexion

**Figure 8:**
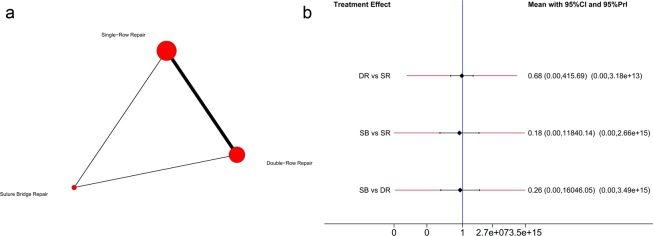
(**a**) Network plot of treatment comparisons for forward flexion. The size of the red area indicates the sample size of each group, and the thickness indicates the results of comparisons between two groups; (**b**). The forest plot and predictive interval plot of network meta-analysis for forward flexion. SR: Single-Row Repair; DR: Double-Row Repair; SB: Suture Bridge Repair.

Regarding forward flexion, six trials^29,32–34,38,44^ were included in this network meta-analysis, and the analysis of the comparisons of forward flexion is illustrated in Fig. 8a. Hierarchies of effect size (MD with 95% CI) based on pairwise meta-analysis is presented in Fig. 7d–f. Hierarchies of effect size (MD with 95% CI and 95% Prl) based on network meta-analysis is presented in Fig. 8b. Both pairwise meta-analysis and network meta-analysis indicated no significant differences among SR repair, DR repair and SB repair in terms of forward flexion. Based on the SUCRA probability, the ranking from first to third was SB repair (0.610), DR repair (0.483) and SR repair (0.408).

### Inconsistency, publication bias and sensitivity analyses

In general, the results based on the pairwise meta-analysis matched well with those based on the network meta-analysis. In this study, no inconsistencies for each outcome was found in the Dias model analysis (Table 3). The publication bias was only detected in the comparison of DR repair versus SR repair for the outcome of forward flexion (Table 4). This may be due to the number of studies included in this analysis was small. Fixed effects and random effects models were compared to detect the sensitivity. The results based on the fixed effects model (effective number of parameters [pD] = 14.96, deviance information criterion [DIC] = 48.88) were similar to those based on the random effects model (pD = 21.85 and DIC = 50.84).

**Table 3:** Dias model analysis for inconsistency of network meta-analysis.

Outcome	Comparsion	Direct		Indirect		Difference		P > |z|
		Coef.	Std. Err.	Coef.	Std. Err.	Coef.	Std. Err.	
Retear rate	SR vs DR	−0.53	0.27	−0.11	0.96	−0.42	0.99	0.675
	SR vs SB	−0.70	0.40	−1.77	0.75	1.08	0.85	0.203
	DR vs SB	−1.13	0.50	0.13	0.48	−1.16	0.69	0.071
Constant score	SR vs DR	−0.06	1.38	2.98	2.80	−3.05	3.12	0.328
	SR vs SB	0.50	1.71	−3.26	2.64	3.76	3.14	0.231
	DR vs SB	−2.67	2.03	0.69	2.25	−3.35	3.03	0.268
External rotation	SR vs DR	−1.45	3.87	6.59	10.04	−8.04	10.76	0.455
	SR vs SB	1	6.93	−7.04	8.24	8.04	10.76	0.455
	DR vs SB	−5.59	7.27	2.45	7.94	−8.04	10.76	0.455
Forward flexion	SR vs DR	−0.32	3.83	−0.89	11.83	0.57	12.44	0.964
	SR vs SB	−2.00	8.51	−1.43	9.07	−0.57	12.44	0.963
	DR vs SB	−1.11	8.22	−1.68	9.34	0.57	12.44	0.964

SR: Single-Row Repair; DR: Double-Row Repair; SB: Suture Bridge Repair.

**Table 4:** Egger’s test for publication bias of pairwise meta-analysis.

Outcome	comparison	Coef.	Std. Err.	t	P > |t|	95% Conf. Interval
Retear rate	DR vs SR	0.25	1.14	0.22	0.834	(−2.39, 2.88)
	SB vs DR	—	—	—	—	—
	SB vs SR	4.13	4.90	0.84	0.488	(−16.97, 25.24)
Constant score	DR vs SR	−1.37	1.51	−0.91	0.404	(−5.25, 2.50)
	SB vs DR	−1.04	1.07	−0.97	0.511	(−14.68, 12.60)
	SB vs SR	−7.63	4.79	−1.59	0.252	(−28.24, 12.99)
External rotation	DR vs SR	−5.07	0.55	−9.24	0.069	(−12.05, 1.90)
	SB vs DR	—	—	—	—	—
	SB vs SR	—	—	—	—	—
Forward flexion	DR vs SR	−10.89	1.13	−9.60	0.011	(−15.76, −6.01)
	SB vs DR	—	—	—	—	—
	SB vs SR	—	—	—	—	—

SR: Single-Row Repair; DR: Double-Row Repair; SB: Suture Bridge Repair.

### Discussion

The network meta-analysis revealed hierarchies based on the primary (retear rate) and secondary outcomes (constant score, external rotation and forward flexion) for patients diagnosed with rotator cuff tears who underwent SR, SB and DR repair. The meta-analysis indicated the following: (1) SR repair resulted in a higher retear rate than DR and SB repairs; (2) the SR and DR repairs had higher incidences of retear than SB repair; (3) the ranking based on the constant score was SB repair, SR repair and DR repair; (4) the ranking for external rotation was SB repair, SR repair and DR repair; and (5) the ranking for forward flexion was SB repair, DR repair and SR repair.

There are some advantages of this type of network meta-analysis: (1) a network meta-analysis provides greater accuracy than a conventional meta-analysis^15^; (2) the network meta-analysis provided indirect comparisons^45^, and SUCRA, which could improve the precision of even slight distinctions between SR, SB and DR repairs.

However, this study also has some limitations. First, some low quality RCTs and case-control studies were included in this study, which may have reduced the significance of the conclusions. Second, the sample size was small, which reduced the accuracy. Third, some potential biases in the data, such as the size of rotator cuff tear, patient age and performance bias, could have affected the accuracy. Finally, some studies did not provide detailed information, such as the standard deviations; therefore, the inadequate data were replaced with statistical methods based on the provided data.

Retear remains a major concern after arthroscopic rotator cuff repair. Hein^10^ previously performed a qualitative review without quantitative analysis to report that both DR repair and SB repair resulted in a lower retear rate than SR repair in most tear size categories, and differences in retear rates were found between DR and SB repair. Spiegl^8^ summarized eight reviews of DR and SR repair and found a higher retear rate following SR repair than after DR repair. Sobhy^46^ also found that SR repair resulted in a higher radiological overall detected retear rate and radiological partial-thickness retear rate than DR repair. Moreover, Bedeir’s study^47^ suggested that DR and SB repair had a higher type 2 retear (medial cuff failure) rate than SR repair. Our network meta-analysis combined indirect and direct evidences to calculate the difference among multiple techniques for rotator cuff repair using quantitative method, which revealed that the DR and SB repairs resulted in lower retear rates than SR repair. Additionally, no statistically significant difference was found between the DR and SB repairs regarding the retear rate. Furthermore, the posterior probabilities and SUCRA were used to differentiate the slight distinctions among SB, DR and SR repair. In terms of achieving lower retear rates, the treatments were ranked as follows: SB repair, DR repair, and SR repair.

The constant score includes shoulder function, range of motion, pain and strength and is used to assess shoulder therapies^48^. In a previous review^8^, no significant difference was found between SR repair and DR repair in terms of the constant score. No previous evidence-based study has analyzed the difference between SB repair and SR or DR repair. In this network meta-analysis, no significant difference was observed among SR repair, DR repair and SB repair in terms of the constant score. Based on the SUCRA, the treatments were ranked as SB repair, SR repair, and DR repair in terms of the achievement of higher constant scores.

The range of motion is critical for assessing the therapeutic value of these three arthroscopic methods. Prasathaporn^14^ previously reported that DR repair provides greater external rotation but found no statistically significant difference in forward flexion. Xu^49^ found no significant differences in external rotation or forward flexion. This network meta-analysis revealed no significant differences among SR repair, DR repair and SB repair in terms of external rotation or forward flexion. Moreover, SB repair provided a greater range of motion (external rotation and forward flexion) than SR and DR repair according to the SUCRA.

In summary, this network meta-analysis provides evidence that SB repairs might be the best choice to improve the postoperative recovery of function and decrease the retear rate because of the greater tendon-bone contact area and the higher tendon-bone contact pressure.

## Supplementary information

Table S1

Table S2

## Data Availability

No datasets were generated or analysed during the current study and all datas come from the listed authors.
