# Supplementary material for: The Clinical Effect of Arthroscopic Rotator Cuff Repair techniques: A Network Meta-Analysis and Systematic Review
Source: Sci Rep. 2019 Mar 11;9:4143. doi: 10.1038/s41598-019-40641-3 (PMC6411857; doi:10.1038/s41598-019-40641-3)
Supplement: Supplementary file 1 — Table S1 [file 41598_2019_40641_MOESM1_ESM.doc]

| **Selection** | **Comparability** | **Exposure** |
| --- | --- | --- |
| 1) Is the case definition adequate? | 1) Comparability of cases and | 1) Ascertainment of exposure |
| a) yes, with independent validation* | controls on the basis of the design | a) secure record (eg: surgical records)* |
| b) yes, eg record linkage or based on self reports | or analysis | b) structured interview where blind to |
| c) no description | a) study controls for __ (Select | case/control status* |
| 2) Representativeness of the cases | the most important factor.)* | c) interview not blinded to case/control |
| a) consecutive or obviously representative series | b) study controls for any | status |
| of cases* | additional factor (This criteria | d) written self report or medical record |
| b) potential for selection biases or not stated | could be modified to indicate | only |
| 3) Selection of Controls | specific control for a second | e) no description |
| a) community controls* | important factor.)* | 2) Same method of ascertainment for cases |
| b) hospital controls |  | and controls |
| c) no description |  | a) yes* b) no |
| 4) Definition of Controls |  | 3) Non-Response rate |
| a) no history of disease (endpoint)* |  | a) same rate for both groups* |
| b) no description of source |  | b) non respondents described |
|  |  | c) rate different and no designation |
|  | | |
| Note: A study can be awarded a maximum of one star for each numbered item within the Selection and Exposure categories. A maximum of two stars can be given for Comparability. | | |

**S1 Table. The Newcastle-Ottawa Scale (NOS) for assessing the quality of case control studies in meta-analyses**

From GA. W, B. S, D. OC, et al. The Newcastle-Ottawa Scale (NOS) for assessing the quality of nonrandomised studies in meta-analyses.
